# Differential gradients of immunotherapy vs targeted therapy efficacy according to the sun-exposure pattern of the site of occurrence of primary melanoma: a multicenter prospective cohort study (MelBase)

**DOI:** 10.3389/fonc.2023.1250026

**Published:** 2023-10-23

**Authors:** David Russo, Stéphane Dalle, Olivier Dereure, Laurent Mortier, Sophie Dalac-Rat, Caroline Dutriaux, Marie-Thérèse Leccia, Delphine Legoupil, Henri Montaudié, Eve Maubec, Julie De Quatrebarbes, Jean-Philippe Arnault, Florence Granel Brocard, Philippe Saïag, Brigitte Dreno, Clara Allayous, Bastien Oriano, Wendy Lefevre, Céleste Lebbé, Lise Boussemart

**Affiliations:** ^1^ Department of Dermatology, Pontchaillou Hospital, CHU de Rennes, Rennes, France; ^2^ Cancer Research Center of Lyon, Hospices Civils de Lyon, Pierre-Bénite, France; ^3^ Department of Dermatology, Hôpital Saint-Eloi, CHU de Montpellier, Montpellier, France; ^4^ Université Lille, Centre Hospitalier Régional Universitaire de Lille, Lille, France; ^5^ Dermatology Department, CHU Dijon Bourgogne, CHU Le Bocage, Dijon, France; ^6^ Centre Hospitalier Universitaire de Bordeaux, Hôpital Saint-André, Bordeaux, France; ^7^ Dermatology Department, CHU Albert Michalon, Grenoble, France; ^8^ Inserm, U1209, Université de Grenoble, Grenoble, France; ^9^ Dermatology Department, CHRU Brest, Brest, France; ^10^ Dermatology Department, Nice Hospital, Nice, France; ^11^ Dermatology Department, Avicenne Hospital, AP-HP, Bobigny, France; ^12^ Dermatology Department, CH d’Annecy, Pringy, France; ^13^ Dermatology Department, CHU Amiens Picardie, Amiens, France; ^14^ Dermatology Department, CHU de Nancy, Nancy, France; ^15^ Dermatology Department, Université de Versailles-Saint Quentin en Yvelines, AP-HP, Boulogne, France; ^16^ Nantes Université, Univ Angers, INSERM, Immunology and New Concepts in ImmunoTherapy, INCIT, UMR 1302, Nantes, France; ^17^ AP-HP, Dermatology Department, Hôpital Saint-Louis, Paris, France; ^18^ Université de Paris, AP-HP Saint-Louis Hospital, Dermatology Department, INSERM U976, Paris, France; ^19^ Dermatology Department, CHU de Nantes, Nantes, France

**Keywords:** melanoma, immunotherapy, sun-exposure, acral melanoma, UV signature

## Abstract

**Background:**

The tumor mutational burden (TMB) is high in melanomas owing to UV-induced oncogenesis. While a high TMB is a predictive biomarker of response to PD-1 inhibitors, it may be associated with the rise of resistant clones to targeted therapy over time. We hypothesized that survivals may depend on both the sun-exposure profile of the site of primary melanoma and the type of systemic treatment.

**Patients and methods:**

Patients were screened from MelBase, a multicenter biobank dedicated to the prospective follow-up of stage III/IV melanoma. All patients with a known cutaneous primary melanoma who received a 1st-line systemic treatment by immunotherapy or targeted therapy were included (2013-2019). Outcomes were progression-free survival (PFS) and overall survival (OS).

**Results:**

973 patients received either anti PD-1(n=466), anti CTLA-4(n=143), a combination of both (n=118), or targeted therapies (n=246). Patients’ characteristics at treatment initiation were: male (62%), median age of 62, AJCC stage IV (84%). Median follow-up was 15.5 months. The primary melanoma was located on chronically sun-exposed skin in 202 patients (G1: head neck), on intermittently sun-exposed skin in 699 patients (G2: trunk, arms, legs), and on sun-protected areas in 72 patients (G3: palms, soles). Median PFS was significantly higher in G1 under anti PD-1 treatment (8.7 months vs 3.3 and 3.4 months for G2 and G3, respectively) (p=0.011). PFS did not significantly differ in other groups. Similarly, median OS was significantly higher in G1 receiving 1^st^ line anti PD-1 treatment (45.6 months vs 31.6 and 21.4 months for G2 and G3) (p=0.04), as opposed to 1^st^ line targeted therapy (19.5 months vs 16.3 and 21.1 months for G1, G2 and G3 respectively).

**Conclusion:**

Our study confirms that immunotherapy with anti PD-1 is particularly recommended for melanomas originating from chronically sun-exposed areas, but this finding needs to be confirmed by further research.

## Background

In Western countries, melanoma holds the unfortunate record for the largest increase in cancer incidence in the past 50 years, doubling every ten years. In Europe, this cancer kills more than 25,000 people annually (1). Almost three quarters of melanomas today affect people over 50 years old.

As with most cancers, the risk factors identified to date can be classified into 2 groups: intrinsic factors, related to the genetic background of the individual, and extrinsic factors, related to the environment. More precisely, most melanomas result from the inability of a skin phototype (intrinsic factor), endowed with a given immune system, to repair the intracellular damage induced by exposure to carcinogenic agents, in particular UV radiation (UVR) (extrinsic factor).

On the therapeutic level, the treatment of advanced or metastatic melanoma has been revolutionized in the last decade, with the advent of immunotherapy with inhibitors of immune checkpoints known as “checkpoint inhibitors”, including anti PD-1 and anti CTLA-4 (2, 3). This new class of treatment has been shown to be effective in several types of skin cancers, commonly secondary to mutagenic UVR, with high tumor mutational burdens (TMB: number of non-synonymous mutations per Megabase of DNA).

In fact, numerous studies have shown the link between TMB and response to anti PD-1 monotherapy, regardless of the histological type of cancer. The high rates of responses to anti PD-1 observed in the context of high mutational loads are attributed to the strong immunogenicity generated by the neoepitopes induced by the numerous mutations (4–6). But not all melanomas are due to UVR, such as plantar or genital melanomas. The chances of response to immunotherapy increase from 40 to 60% when combining anti PD-1 to anti CTLA-4, but at the cost of high and potentially durable toxicities (3).

In contrast, a high TMB has been shown to be negatively associated with clinical outcomes in metastatic lung cancer patients treated with targeted therapy such as EGFR-TKI (7). In the context of targeted therapy, which is the main other potentially effective therapeutic class available in advanced melanoma (8), a high TMB may also favor an increased pace with which a resistant subclone would, under the selective pressure of targeted therapy, lead to clinical resistance. However, this has never been demonstrated in melanoma.

Thus, we recently showed that TMB can help guiding the best treatment choice for each patient (9). Unfortunately, TMB measurement is not accessible to every patient, due to its cost and the technologies it requires. But in our recent study, we have shown that high sun exposure skin areas usually give rise to highly mutated melanomas and strong UV signature, as opposed to sun protected areas.

In case of advanced melanoma, molecular biology platforms perform targeted sequencing of certain exons of the *BRAF, KIT* and *NRAS* genes in the tumor DNA. Techniques, sensitivity and depth of coverage vary from platform to platform. Wide coverages make it possible to determine the TMB by extrapolation, but unfortunately, the cost of assessing the TMB is significant and this data is not accessible to everyone (10). Moreover, in the absence of a standardized technique, a threshold response value to immunotherapy in relationship to TMB, is lacking.

Here, because high skin cancer TMB often results from cumulative UV-exposure over lifetime, we hypothesized that the sun-exposure pattern of the site of the formerly excised primary melanoma could influence survivals following first-line treatment by either immunotherapy or targeted therapy.

## Patients and methods

### Design, population and endpoints

We studied the French multicentric MelBase prospective cohort of unresectable stage III or IV cutaneous melanoma. We included all patients, who received a first-line systemic treatment by immunotherapy (nivolumab, pembrolizumab, ipilimumab alone, or the combination of both nivolumab and ipilimumab) or combined anti BRAF and anti MEK targeted therapy (dabrafenib and trametinib or vemurafenib and cobimetinib) between January 2013 and November 2019. Patients with uveal, mucosal or unknown primary melanoma were excluded.

According to the location of the known cutaneous primary melanoma, we allocated each patient to a group of Bastian BC’s sun-exposure patterns (11, 12): Group #1 (G1) included patients whose melanoma originated from a chronically sun-exposed area such as head and neck, Group #2 (G2) included patients whose melanoma originated from an intermittently sun-exposed area such as trunk, arms and legs, and Group #3 (G3) included patients whose melanoma originated in sun-protected areas such as palms and soles. Primary outcome was progression-free survival (PFS), secondary outcome was overall survival (OS).

### Statistical analysis

The follow-up duration median and range were calculated. The median PFS and OS values were evaluated using the Kaplan–Meier method and Cox proportional hazards regression models. The PFS and OS values across the subgroups were compared using the log-rank test in case of non-proportionality in Cox’s model or using Fleming-Harrington estimator if not.

Using univariate and multivariable cox proportional hazard models, we analyzed the associations between groups and PFS. In the multivariate analysis, we adjusted for age, Breslow (<1mm, 1-2mm, 2-3mm, 3-4mm, >4mm), ECOG performance status, BRAF status (wild type vs mutated), LDH level (normal vs elevated), metastatic location (brain metastasis, cutaneous, liver, lung, lymph node). To correct the non-proportional hazards assumptions, a piecewise modeling has been made.

All analyses were carried out using R statistical software version 3.5.2 (The R Foundation for Statistical Computing, Vienna, Austria).

## Results

### Population

973 patients were included in our study. Patients’ characteristics at treatment initiation are shown in **Table 1**: male gender (62%), median age of 62, ECOG 0-1 (84%) and normal LDH (52%). Median follow-up was 15.5 months.

**Table 1:** Demographic and clinical characteristics at baseline.

	Characteristics	N (%)	G1	G2	G3
SEX	f	372 (38)	57 (28)	274 (39)	41 (57)
	m	601 (62)	145 (72)	425 (61)	31 (43)
AGE	18-40	113 (12)	25 (12)	87 (12)	1 (1)
	40-65	427 (44)	72 (36)	320 (46)	35 (49)
	65*<*	433 (45)	105 (52)	292 (42)	36 (50)
	median Q1-Q3	62.0 (49.0 to 71.0)	65.5 (52.0 to 74.8)	61.0 (48.0 to 71.0)	64.5 (53.8 to 71.2)
LOCATION	Trunk	378 (39)	0 (0)	378 (54)	0 (0)
	Head & neck	202 (21)	202 (100)	0 (0)	0 (0)
	Leg	113 (12)	0 (0)	113 (16)	0 (0)
	Arm	105 (11)	0 (0)	105 (15)	0 (0)
	Thighs	78 (8)	0 (0)	78 (11)	0 (0)
	Sole/foot	66 (7)	0 (0)	0 (0)	66 (92)
	Forearm	25 (3)	0 (0)	25 (4)	0 (0)
	Palm/hand	6 (1)	0 (0)	0 (0)	6 (8)
BRESLOW (mm)	*<*1	112 (12)	22 (11)	87 (12)	3 (4)
	1-2	247 (25)	44 (22)	192 (27)	11 (15)
	2-3	144 (15)	29 (14)	102 (15)	13 (18)
	3-4	142 (15)	20 (10)	111 (16)	11 (15)
	4*<*	286 (29)	70 (35)	184 (26)	32 (44)
	Unknown	42 (4)	17 (8)	23 (3)	2 (3)
	Median Q1-Q3	3.0 (1.5 to 5.4)	3.5 (1.7 to 6.0)	3.0 (1.5 to 5.0)	4.0 (2.3 to 6.4)
ULCERATION	No	471 (48)	113 (56)	339 (48)	19 (26)
	Yes	430 (44)	72 (36)	310 (44)	48 (67)
	Unknown	72 (7)	17 (8)	50 (7)	5 (7)
HISTOLOGY	SSM	503 (52)	81 (40)	404 (58)	18 (25)
	NM	242 (25)	42 (21)	190 (27)	10 (14)
	Unclassable	69 (7)	22 (11)	41 (6)	6 (8)
	Unknown	56 (6)	12 (6)	42 (6)	2 (3)
	ALM	39 (4)	1 (0)	3 (0)	35 (49)
	LMM	30 (3)	29 (14)	1 (0)	0 (0)
	Desmoplastic	9 (1)	5 (2)	4 (1)	0 (0)
	MLM	7 (1)	6 (3)	1 (0)	0 (0)
	Spitzoid	7 (1)	2 (1)	4 (1)	1 (1)
	Naevoid	6 (1)	1 (0)	5 (1)	0 (0)
	Ambiguous	5 (1)	1 (0)	4 (1)	0 (0)
ECOG	0	621 (64)	128 (63)	447 (64)	46 (64)
	1	196 (20)	41 (20)	136 (19)	19 (26)
	2	47 (5)	12 (6)	32 (5)	3 (4)
	3	15 (2)	3 (1)	12 (2)	0 (0)
	4	2 (0)	0 (0)	2 (0)	0 (0)
	Unknown	92 (9)	18 (9)	70 (10)	4 (6)
	Median Q1-Q3	1.0 (1.0 to 2.0)	1.0 (1.0 to 2.0)	1.0 (1.0 to 2.0)	1.0 (1.0 to 2.0)
LDH	High	251 (26)	49 (24)	192 (27)	10 (14)
	Normal	509 (52)	103 (51)	362 (52)	44 (61)
	Unknown	213 (22)	50 (25)	145 (21)	18 (25)
AJCC	0	5 (1)	3 (1)	2 (0)	0 (0)
	IA	1 (0)	1 (0)	0 (0)	0 (0)
	IIB	1 (0)	1 (0)	0 (0)	0 (0)
	IIC	1 (0)	1 (0)	0 (0)	0 (0)
	IIIA	1 (0)	0 (0)	1 (0)	0 (0)
	IIIB	24 (2)	3 (1)	20 (3)	1 (1)
	IIIC	123 (13)	28 (14)	75 (11)	20 (28)
	IV M1a	88 (9)	14 (7)	62 (9)	12 (17)
	IV M1b	172 (18)	44 (22)	116 (17)	12 (17)
	IV M1c	557 (57)	107 (53)	423 (61)	27 (38)

They received either immunotherapy (n=727), or targeted therapy (n=246). Immunotherapy consisted in anti PD-1 monotherapy (n=466), including either nivolumab (n=171) or pembrolizumab (n=295) alone, or anti CTLA-4 alone (n=143), or a combination of nivolumab and ipilimumab (n=118). Targeted therapy consisted in the combination of dabrafenib and trametinib (n=187) or vemurafenib and cobimetinib (n=59).

The primary melanoma arose on chronically sun-exposed skin in 175 patients (G1: head neck), on intermittently sun-exposed skin in 615 patients (G2: trunk, arms, legs), and on sun-protected areas in 65 patients (G3: palms, soles). The treatment received by each group of patients are detailed in **Table 2**.

**Table 2:** First-line systemic treatments delivered according to sunexposure groups.

	G1	G2	G3	Total
aPD1	111 (55)	318 (45)	37 (51)	466 (48)
Ipilimumab	22 (11)	101 (14)	20 (28)	143 (15)
TT (targeted therapy)	42 (21)	196 (28)	8 (11)	246 (25)

The most frequent histologic types were SSM (52%) and nodular melanoma (NM) (25%). As expected, LMM (n=30) were only found on G1, and ALM (n=39) were predominant in G3. Median Breslow was 3mm, with no significant difference between sun exposure groups. The majority of patients were treated for stage IV melanoma (84%).

Fifty-eight percent of patients were BRAF wild type. BRAFV600 mutations were detected among 37% of patients (30% of G1 patients, 41% of G2 patients, and 15% of G3 patients).

### Survival analysis

**Figure 1:**
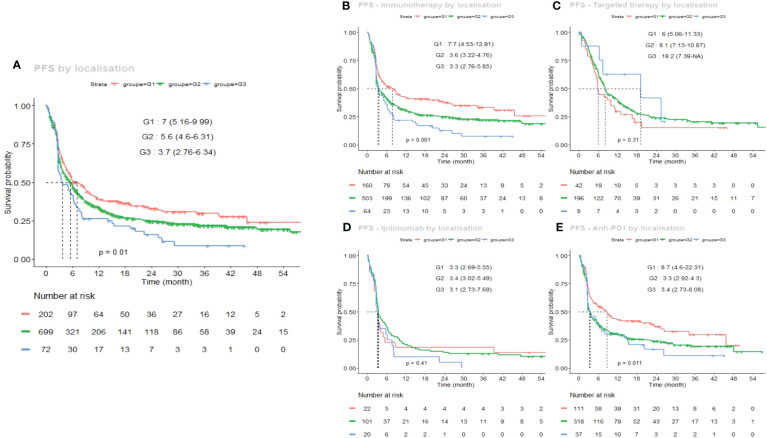
Kaplan–Meier estimates of progression free survival (PFS) according to the sun-exposure groups G1 (chronically sun-exposed, red curve), G2 (intermittently sun-exposed, green curve) and G3 (sun-protected, blue curve). **(A)** global PFS curves regardless of the first-line systemic treatment; **(B)** under immunotherapy (anti PD-1 or anti CTLA-4), **(C)** under targeted therapy (dabrafenib and trametinib or vemurafenib and cobimetinib); **(D)** under ipilimumab alone; **(E)** under anti PD-1 alone (nivolumab or pembrolizumab).

The Kaplan–Meier PFS curves for each sun-exposure group, in the total population, and for each treatment, is shown in **Figures 1A–E**. Regardless of the first-line treatment, median PFS significantly varied between groups, ranging from 7 months for G1, to 5.6 months for G2 and 3.7 months in G3 (**Figure 1A**, p=0.01).

This better PFS in G1 was even greater under immunotherapy (**Figure 1B**), particularly under anti PD-1 monotherapy (**Figure 1E**: 8.7 months for G1 vs 3.3 and 3.4 months for G2 and G3, respectively) (p=0.011). This G1 advantage was not significant under ipilimumab (**Figure 1D**). On the other hand, the median PFS was long in G3 under targeted therapy (19.2 months vs 8.1 and 6 months for G2 and G1) (p=0.31), but this was not significantly different due to the relatively low number of patients treated with targeted therapy in G3 (n=8).

**Figure 2:**
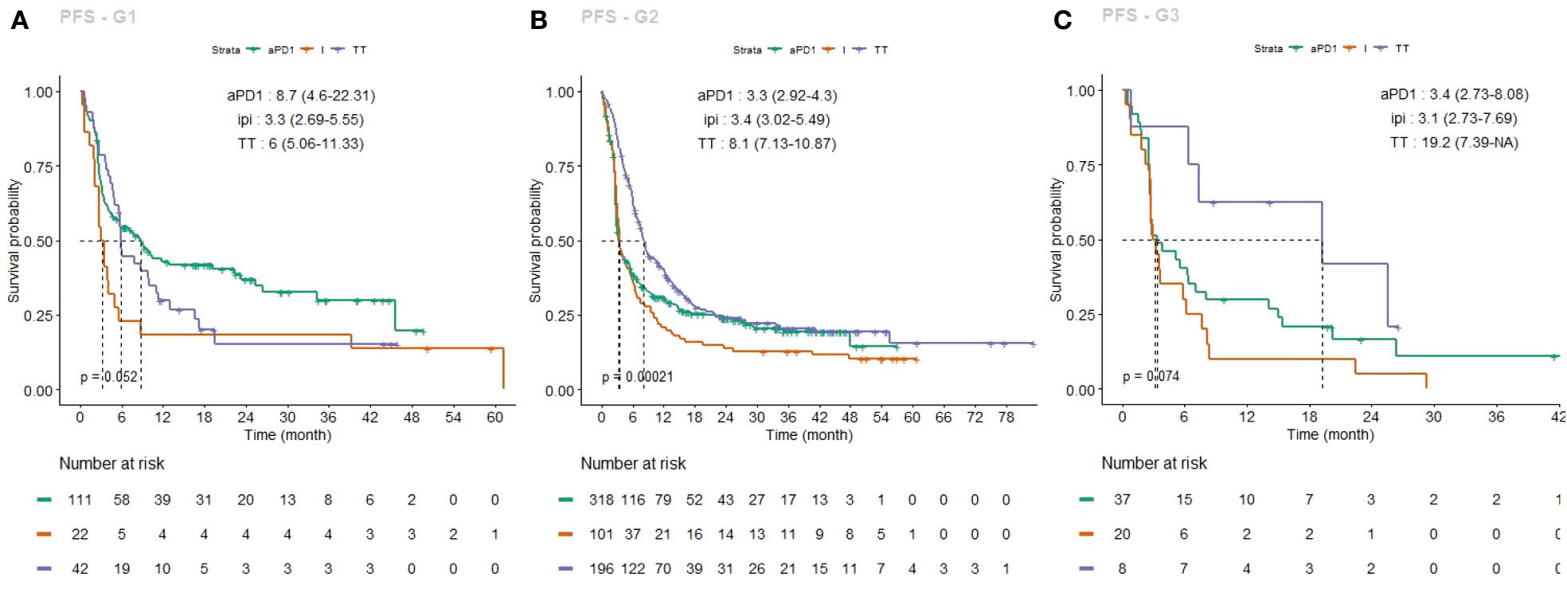
Kaplan–Meier estimates of progression free survival (PFS), each group taken individually, under each first-line treatment (PD-1 inhibitors, green curve; ipilimumab, light brown curve; targeted therapy, purple curve). **(A)** G1 PFS curves; **(B)** G2 PFS curves, **(C)** G3 PFS curves.

To facilitate multimodal comparisons, differential PFS curves depending on first-line treatment are also shown in **Figure 2**, separately for each group (A: G1, B: G2, C: G3).

Multivariate analysis also confirmed a significant association between PFS and LDH rate (HR 1.47, CI95% (1.23-1.75), p<0.001), ECOG status (HR 0.52, CI95% (0.4-0.68) p<0.001), and the presence of brain metastasis (HR 1.47, CI95% (1.16-1.68) p<0.001).

**Figure 3:**
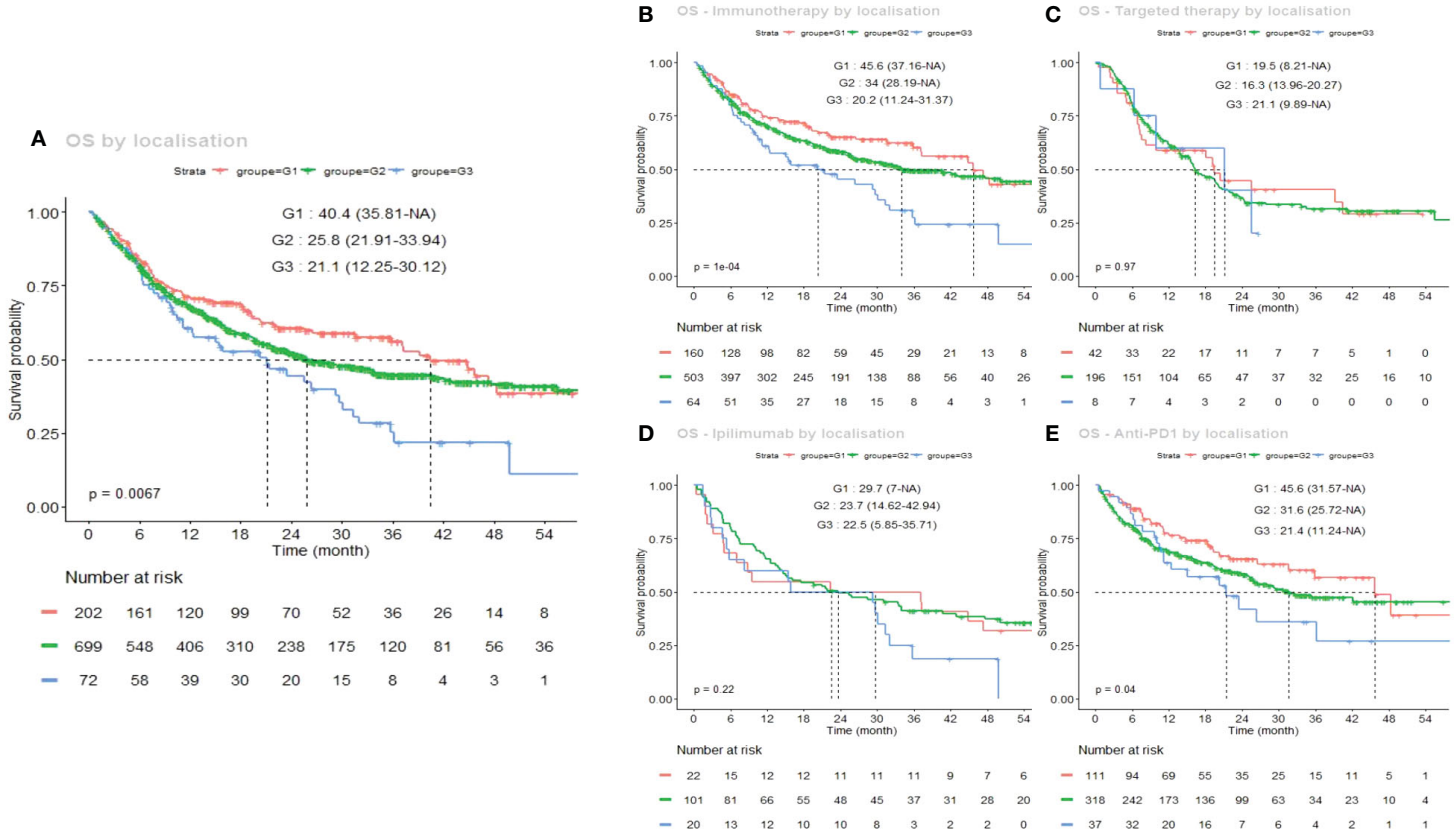
Kaplan–Meier estimates of overall survival (OS) according to the sun-exposure groups G1 (chronically sun-exposed, red curve), G2 (intermittently sun-exposed, green curve) and G3 (sun-protected, blue curve). **(A)** global OS curves regardless of the first-line systemic treatment; **(B)** under immunotherapy (anti PD-1 or anti CTLA-4), **(C)** under targeted therapy (dabrafenib and trametinib or vemurafenib and cobimetinib); **(D)** under ipilimumab alone; **(E)** under anti PD-1 alone (nivolumab or pembrolizumab).

The Kaplan–Meier OS curves for each sun-exposure group, in the total population, and for each treatment, is shown in **Figures 3A–E**. Similarly to PFS, regardless of the first-line treatment, median OS varied significantly between groups, ranging from 40.4 months for G1, to 25.8 months for G2 and 21.1 months in G3 (**Figure 2A**, p=0.0067). Here again, this better OS in G1 was even more significant under immunotherapy (**Figure 3B**), particularly under first-line anti PD-1 monotherapy (**Figure 3E**: 45.6 months for G1 vs 31.6 and 21.4 months for G2 and G3) (p=0.04).

**Figure 4:**
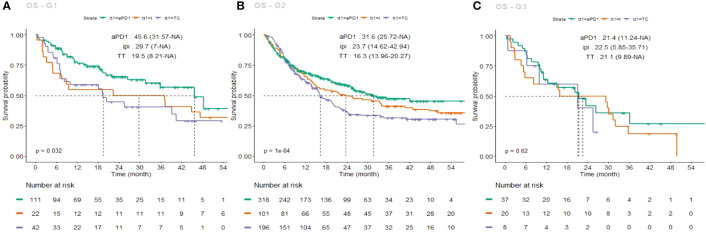
Kaplan–Meier estimates of overall survival (OS), each group taken individually, under each first-line treatment (PD-1 inhibitors, green curve; ipilimumab, light brown curve; targeted therapy, purple curve). **(A)** G1 OS curves; **(B)** G2 OS curves, **(C)** G3 OS curves.

Differential OS curves depending on first-line treatment are also shown in **Figure 4**, separately for each group (A: G1, B: G2, C: G3).

Finally, due to the very low number of patients, the combination of ipilimumab and nivolumab PFS and OS curves are shown separately in **Supplementary Figure 1**, only for G1 and G2. The median PFS for G1 is not reached yet, but the G2 PFS reaches 9 months, which is better than the best median PFS observed for this group in **Figure 2B** (targeted therapy: 8.1 months). Although these preliminary curves are promising, due to the very low number of events, we cannot conclude on this recent combined immunotherapy yet.

## Discussion

Immunotherapy and combined targeted therapies have proven successful, as they produce a significant clinical benefit in a subset of unresectable melanoma patients. However, identification of the best treatment sequence, especially in case of the presence of a BRAF V600 mutation, remains a challenge. Indeed, the BRAFV600E driver mutation being devoid of any “UV-signature”, it can occur in colon or thyroid cancers and its presence is not particularly linked to a high TMB or response to immunotherapy.

Here, we showed that the more sun-exposed the skin area where the primary melanoma arose, the more likely the patient is to benefit from a first-line treatment with immunotherapy, particularly PD-1 inhibitor alone, both in terms of PFS and OS. This has been confirmed in another recent study by Liu et al (11), restricted to immune checkpoint inhibitors. Beyond the strict location of the primary melanoma, visible photoaging could efficiently help identifying patients who will benefit from PD-1 inhibitors as monotherapy. On the other hand, our hypothesis that lower TMB in melanomas arising on sun protected areas (G3) would trigger better outcomes under targeted therapy, has not been confirmed probably because of the low number of patients in this group. In addition, when interpreting G3 OS, one should keep in mind that palms and soles melanomas often evolve without causing deadly metastases for longer than other types of melanomas.

To our knowledge, this is the largest cohort focusing on the response profile to systemic treatments according to the location of the primary melanoma.

Numerous studies have associated high TMBs with clinical benefit from immunotherapy. However, the value of TMB is heterogeneous among all melanomas, and measurement techniques are not standardized. Chronically sun-damaged (CSD) melanomas are considered to have the highest TMB (25mut/Mb) as compared to average 15mut/Mb reported in non-CSD melanomas. Shain et al. hypothesized that these differences depend on UV exposure pattern with a chronic sun exposure and solar elastosis associated with higher TMBs (12).

Moreover, together with DNA damage/mutations, sun-exposure induces inflammation that probably favors immunotherapy approaches.

The advent of targeted therapies and immunotherapy in the adjuvant and neo-adjuvant settings brings new issues such as which treatment should be favored in the context of BRAF mutated melanomas (3, 13, 14). According to ASCO 2023 guidelines (15), nivolumab plus ipilimumab followed by nivolumab is now preferred over BRAF/MEK inhibitor therapy for patients with unresectable or metastatic cutaneous melanoma—regardless of *BRAF* mutation status or TMB.

We believe that the sun-exposure pattern of the primary melanoma, probably correlated with TMB, which is not broadly available yet, should be integrated as a useful clinical parameter in future guidelines.

In conclusion, our study suggests, that the sun-exposure pattern of the site of occurrence of the primary melanoma can significantly and differentially influence the PFS and OS under PD-1 inhibitors and targeted therapy. In the future, it may also help treatment decisions for any UV-induced skin cancer possibly treated with PD-1/PD-L1 inhibitors vs targeted therapy.

## Data availability statement

The raw data supporting the conclusions of this article will be made available by the authors, without undue reservation.

## Ethics statement

The MelBase protocol was approved by French ethics committee (Comité de Protection des Personnes, Île-de-France XI, No. 12027, 2012). The studies were conducted in accordance with the local legislation and institutional requirements. The participants provided their written informed consent to participate in this study.

## Author contributions

DR and LB designed and analyzed the study. LB wrote the manuscript. SD, OD, LM, SD-R, CD, M-TL, DL, HM, EM, JQ, J-PA, FG, PS, and BD are the clinicians who included patients in this cohort and collected the data. CA, BO, and WL performed statistical analysis. CL and LB supervised the study. All authors contributed to the article and approved the submitted version.

## Supplementary material

Supplementary Figure 1 Kaplan–Meier estimates of progression free survival (PFS: **(A, B)** and overall survival (OS: **(C, D)**, under first-line combined ipilimumab and nivolumab, for G1 and G2.
