# Supplementary figures and images for: Differential gradients of immunotherapy vs targeted therapy efficacy according to the sun-exposure pattern of the site of occurrence of primary melanoma: a multicenter prospective cohort study (MelBase)
Source: Front Oncol. 2023 Oct 23;13:1250026. doi: 10.3389/fonc.2023.1250026 (PMC10627180; doi:10.3389/fonc.2023.1250026)

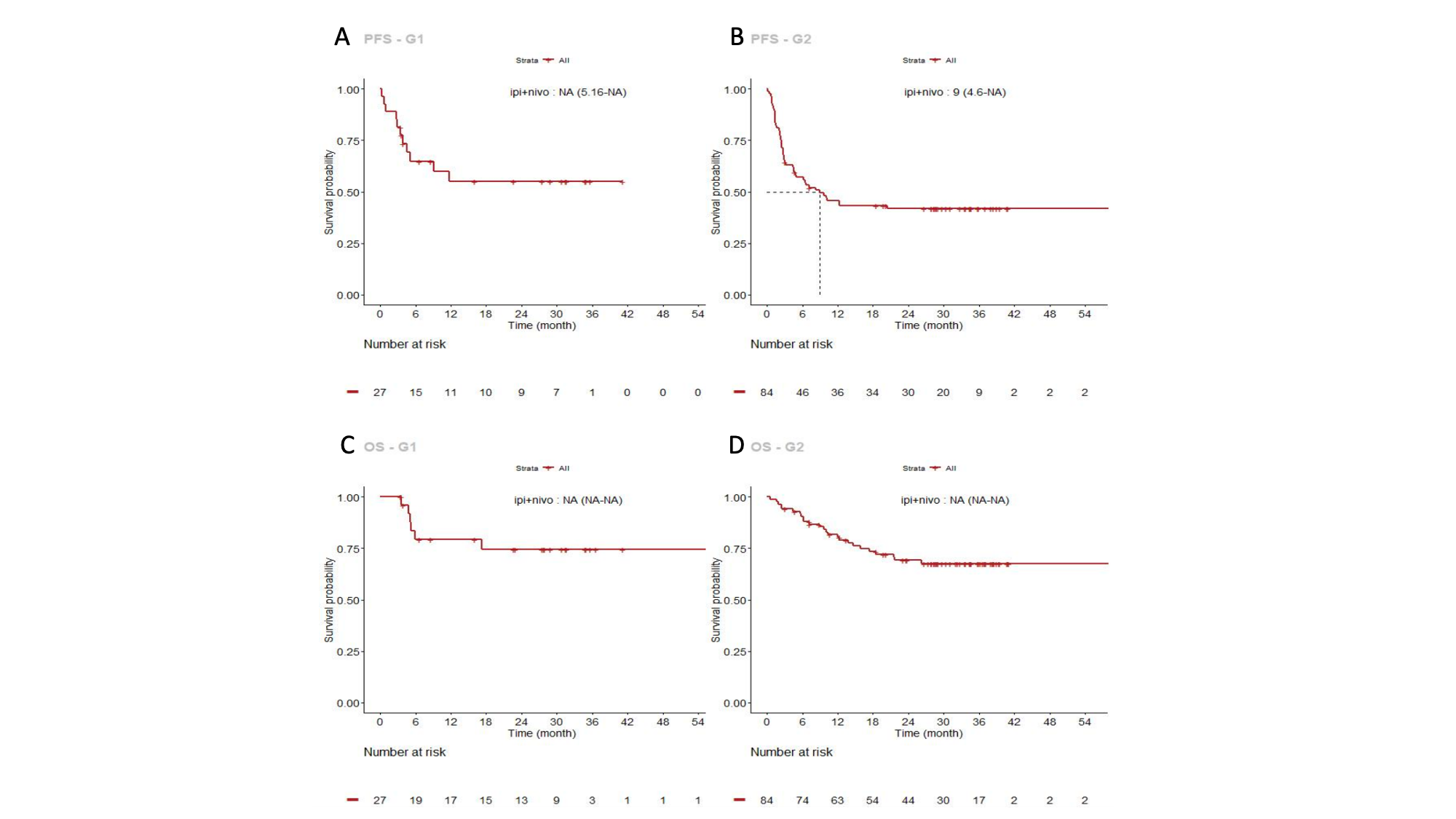

Supplement: Supplementary Figure 1 — Kaplan–Meier estimates of progression free survival (PFS: (A, B) and overall survival (OS: (C, D), under first-line combined ipilimumab and nivolumab, for G1 and G2. [file Image_1.tiff]
